# Biomimetic Membranes with Transmembrane Proteins: State-of-the-Art in Transmembrane Protein Applications

**DOI:** 10.3390/ijms20061437

**Published:** 2019-03-21

**Authors:** Hyunil Ryu, Ahmed Fuwad, Sunhee Yoon, Huisoo Jang, Jong Chan Lee, Sun Min Kim, Tae-Joon Jeon

**Affiliations:** 1 Department of Biological Engineering, Inha University, Incheon 22212, Korea; hyunil.ryu@gmail.com (H.R.); yoonsh0912@gmail.com (S.Y.); huisoojang@gmail.com (H.J.); 12140664@inha.edu (J.C.L.); 2 Biohybrid Systems Research Center, Inha University, Incheon 22212, Korea; ahmedsunny41@gmail.com; 3 Department of Mechanical Engineering, Inha University, Incheon 22212, Korea

**Keywords:** biomimetic membranes, transmembrane proteins, aquaporin, biosensors, DNA sequencing, drug screening, ATP

## Abstract

In biological cells, membrane proteins are the most crucial component for the maintenance of cell physiology and processes, including ion transportation, cell signaling, cell adhesion, and recognition of signal molecules. Therefore, researchers have proposed a number of membrane platforms to mimic the biological cell environment for transmembrane protein incorporation. The performance and selectivity of these transmembrane proteins based biomimetic platforms are far superior to those of traditional material platforms, but their lack of stability and scalability rule out their commercial presence. This review highlights the development of transmembrane protein-based biomimetic platforms for four major applications, which are biosensors, molecular interaction studies, energy harvesting, and water purification. We summarize the fundamental principles and recent progress in transmembrane protein biomimetic platforms for each application, discuss their limitations, and present future outlooks for industrial implementation.

## 1. Introduction

For several billion years, cells have evolved to be remarkably efficient and highly selective biological units. Therefore, it is difficult to develop materials with performance higher than that of natural biomaterials; thus, the direct use of biomaterials is a reasonable strategy. Researchers have utilized biomaterials, including oligonucleotides [1], enzymes [2], antibodies [3], phospholipids [4], and bioproducts [5], in various studies and applications during the last fifty years. Membrane proteins (MPs) are biomaterials with unique characteristics that have high specificity and selectivity yield, but research has been hampered by limitations associated with the difficulty of incorporating them into amphiphilic housing or a lipid bilayer membrane [6,7]. As a result, the number of MPs with known 3D structure and function is very small compared to the total number of MPs, mainly due to the limitations of X-ray crystallography and purification techniques [6,8]. On the other hand, the investigation of soluble proteins was tremendously promoted by the continuous development of various assays and instrumentation [9]. Among MPs, transmembrane proteins (TMPs) are particularly attracting attention for practical applications because they cross the whole membrane, unlike monotopic proteins that are attached to only one side of the membrane. The TMPs confer unique characteristics on the membrane, including signal transduction and the transport of ions or small molecules across the membrane. Some TMPs bind to the receptors of hormones or neurotransmitter molecules and change their structure by triggering a unique response. Moreover, through active or inactive transport, they selectively transfer substances, such as ions or molecules, across the membrane, thereby generating gradients in substance concentration or energy potential between the intracellular and extracellular environment. Because these properties cannot be reproduced by other molecules, TMPs are being studied as essential biomaterials in diverse sensor, screening, water purification, and energy harvesting applications.

**Figure 1:**
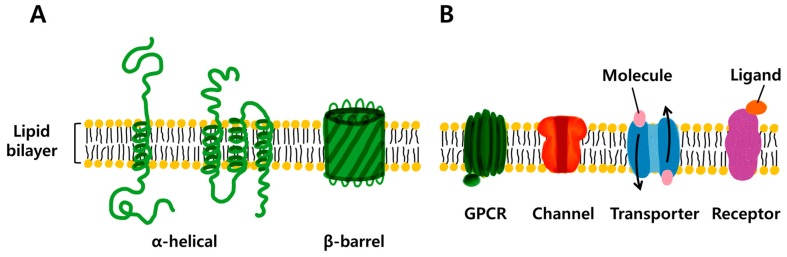
(**A**) Structure of alpha-helical and beta-barrel transmembrane proteins. (**B**) Schematic example of transmembrane protein; G-protein coupled receptors (GPCRs), ion channel, transporter, receptor.

Due to the amphiphilic nature of TMPs, they form either alpha-helical or beta-barrel structures while embedded in the membrane. Alpha-helical TMPs are found in the inner membranes of bacterial cells or in the plasma and outer membrane of eukaryotes. In contrast, beta-barrel TMPs have been found only in the outer membrane of Gram-negative bacteria, the cell walls of Gram-positive bacteria, and the outer membrane and the chloroplasts of mitochondria [10]. Most alpha-helical TMPs cannot retain their 3D structure and precipitate under hydrophilic conditions, but beta-barrel TMPs can easily be stored in aqueous solution [11]. Therefore, beta-barrel TMPs are much easier to purify and use for different applications [12,13]. Figure 1A shows the alpha-helical or beta-barrel structure in the lipid bilayer. TMPs can be categorized into G-protein coupled receptors (GPCRs), ion channels, carriers and transporters, and other receptors (Figure 1B). Since GPCRs are the most important molecules for human signal transduction, they are widely used as a target for drug screening. Potassium or sodium channels are also used in basic research and as screening tools in the pharmaceutical industry. Relatively small ion channel-forming proteins that are usually shorter than 100 amino acids such as gramicidin, or beta-barrel ion channels such as hemolysin are used for applications in ionic and molecular recognition [14,15], as their synthesis and reconstitution into membranes are comparatively easy. Among diverse selective pore proteins, aquaporin proteins are employed as molecular filters for purifying bulk amounts of water molecules [16]. Rhodopsin, photosystem I, II, and ATPase have been studied to develop an energy harvesting system using purple membrane derived from cytoplasmic membrane and containing TMPs for photosynthesis to use their high energy conversion efficiency, even though their purification and reconstruction are difficult [17,18,19,20].

**Figure 2:**
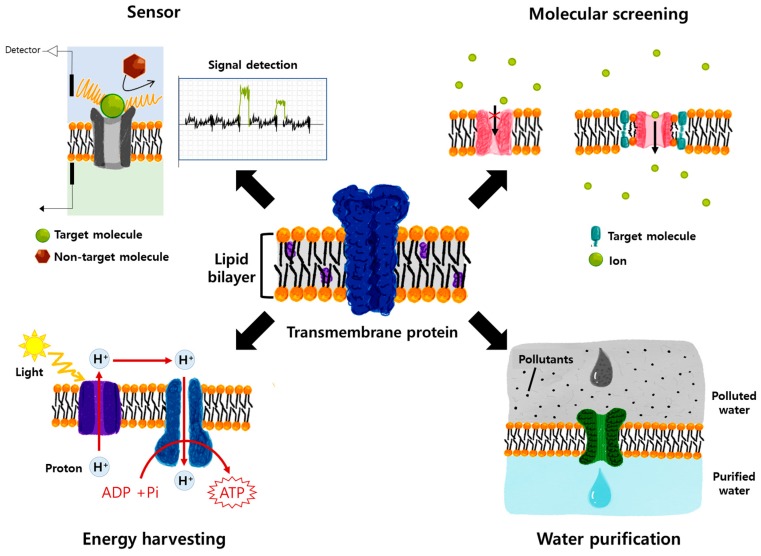
Representative schematic image of potential applications of transmembrane proteins: small molecule sensor, drug and membrane/membrane protein interaction studies, energy harvesting, and water purification platform.

This review will provide a brief overview of different MPs being studied for practical applications. The first chapter will focus on biosensor development utilizing the high selectivity of MPs. In the second chapter, we will concisely review drug screening tools based on MPs, and the third section of this paper will provide a brief overview of energy harvesting applications using biological MPs. In the last section, we will discuss water purification applications using biological water channels (aquaporins). This review will provide basic knowledge about TMPs and their potential use for practical applications to new researchers while providing future insight into the future trends and applications of TMPs. Figure 2 shows the different applications of TMPs.

## 2. Potential Applications of Transmembrane Proteins

### 2.1. Membrane Protein (MP)-Based Biosensor System

Membrane proteins (MPs) have attracted great interest for biosensor applications due to their unique nanostructure and functions. To date, a number of researchers have reported high-sensitivity biosensors that exploit the inherent structure and function of MPs [21]. In this chapter, we summarize the biosensing applications of TMPs in terms of their nanopore structure and receptor-mediated ion transfer.

#### 2.1.1. Biological Nanopore Sensors

In the last two decades, nanopore sensors have emerged as a powerful tool and have had a strong impact on science and biotechnology. Nanopore technology is commonly divided by its materials into biological nanopores and solid-state nanopores. Solid state nanopores are conventionally fabricated by drilling a nanoscopic pore using semiconductor or microfluidic techniques like ion or electron beam sculpting in silicon or graphene-based membranes such as Si, SiN, or SiO_2_. However, most nanopore applications, such as DNA sequencing, small molecule sensing, drug screening, molecular sieving, and biomolecular analysis, require high-precision geometry, sensitivity, and reproducibility, which cannot be achieved with solid-state pores. As our review focuses only on MPs, we will only show biological nanopores, but for interested readers, there are several outstanding reviews on nanopore applications addressing both solid-state and biological nanopore applications and their comparison [22,23,24].

**Figure 3:**
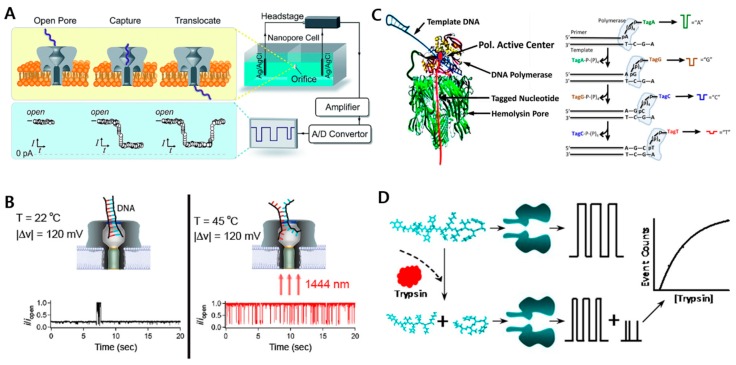
Shows the biological nanopore sensor experimental setup. (**A**) The generic experimental setup for single-molecule studies using a nanopores [25]. (Reproduced with permission from Ying et al. Analyst 2014. Copyright© 2014 Royal Society of Chemistry) (**B**) The experimental setup and analysis of DNA unzipping at elevated temperature by using short-wavelength infrared (SWIR) heating methodology [32]. (Reproduced with permission from Christopher E. Angevine et al. Anal. Chem. 2016. Copyright© 2016 American Chemical Society) (**C**) A design for nanopore sensors with phi29 DNA polymerase molecules attached to the α-Hemolysin (α-HL) pore. The next image shows the sequential detection of tagged nucleotides using sequencing by synthesis (SBS) methodology [37]. (Reproduced with permission from C.W. Fuller et al. PNAS 2016. Copyright© 2016 National Academy of Sciences) (**D**) An indirect strategy to analyze the protein activity. The current before and after trypsin activity is compared [42]. (Reproduced with permission from S. Zhou et al. ACS Sens. 2016. Copyright© 2016 American Chemical Society.

A typical experimental setup consists of two compartments of chambers containing buffer and separated by a 10–100 µm thin Teflon or polyethylene film. Each compartment is connected to the current amplifier through electrodes, and the current across the nanopores is measured by the amplifier [25]. The whole setup is shown in Figure 3A. While measuring currents, the blockage events of analytes that are passing through the nanopore are analyzed on the basis of three different parameters: current amplitude, event duration, and time interval between blockage events.

α-Hemolysin (α-HL) is the most widely used biological nanopore for single-molecule analysis, mainly due to its small inner diameter (~1.4 nm) and structural reproducibility. Initially, α-HL was used for detecting nucleic acids by the electrophoretic transport of ssDNA and ssRNA [26]. As each strand enters the nanopore, four different bases in the ssDNA or ssRNA give their unique current changes. This remarkable concept later enabled several studies to understand the various physical and chemical characteristics of ssDNA by determining their sequence information during translocation [27], RNA diblock copolymer differentiation [28] and ssDNA homopolymer composition [29]. For the accurate reading of each nucleotide, the Bayley group engineered truncated barrel mutants (TBMs) of α-HL with a shorter barrel length than that of WT α-HL, which enables the identification of four mononucleotides using cyclodextrin (CD) adapters, giving rise to better results [30]. However, double-stranded DNA (dsDNA) translocation is currently impossible to measure directly through the α-HL nanopore, mainly because the pore size of α-HL (~1.4 nm) is smaller than the diameter of dsDNA. To make the α-HL nanopore platform more practical, many different methodologies have been employed. To melt dsDNA at an elevated temperature, Angevine et al. developed a short-wavelength infrared (SWIR) heating system that used a laser with local plasmonic heating and direct absorption [31,32]. Briefly, a 1444 nm diode light was focused on the bilayer containing α-HL. Laser elevates solution temperature, and DNA starts unzipping. The unzipped DNA consequently starts translocating through the pore. Figure 3B shows the experimental setup of the system [32]. Further studies analyzed the effect of temperature and electrolyte concentration on the blockage current to understand the current signature in an optimized condition [33,34,35].

The most important and most highly desired application of nanopores is DNA sequencing. However, the major problems associated with the very high translocation speed of DNAs through nanopores (several nucleotides pass through the nanopore in a few micro seconds), resulting in few data points for each base, which hinders further analysis of data. Several methodologies have been adopted to slow the translocation speed, such as low-temperature analysis and changing the solution composition (such as adding salts or optimizing viscosity). The major breakthrough was made in 2012 by Kumar et al. who proposed the nanopore-based sequencing by synthesis (Nano-SBS) platform [36]. Briefly, four nucleotides were labeled with different sizes of tags that are released during the polymerase reaction and enter the nanopore, generating unique current events due to their unique chemical structures. In another strategy, phi29 DNA polymerase was covalently attached to the entrance of the α-HL nanopore. During the synthesis reaction, the PEG polymer tags were moved across the pore, thus creating unique blockage signals [37]. A schematic of the process is shown in Figure 3C.

Another challenge in using protein nanopores for biosensing applications is to identify the amino acid sequences of proteins, which will greatly help drug screening and protein engineering fields. However, again, the fact that polypeptides are greater in diameter than nanopores precludes the direct analysis of protein sequences. Therefore, several indirect methodologies have been reported to analyze proteins using biological nanopores [38,39]. One of the methods proposed is to attach an aptamer at the lumen of the α-HL nanopore, which changes the detection region from the pore to the surface [40,41]. In another approach, trypsin activity was indirectly analyzed by monitoring the current blockage events during the proteolytic cleavage of a lysine-containing peptide. The current modulation resulting from substrate degradation indicates the level of trypsin activity [42]. The process is shown in detail in Figure 3D.

Although α-HL is the most widely utilized biological nanopore for studies of small molecules at the single-molecule level, it still has inherent structural limitations, giving rise to the need for new nanopores. A number of different biological nanopores have been reported and utilized in different applications to enhance their selectivity, sensitivity, and usability, including mycobacterium smegmatis porin A (MspA), Aerolysin (AeL), Phi29 Motor, Cytolysin A (ClyA), and Outer membrane protein G (OmpG). Except for ClyA and phi29, each of these molecules has a smaller pore size than α-HL and cannot be employed for dsDNA or other molecules larger than the pores. Each TMP pore has its own unique structural characteristics, which we summarize in Table 1. On the basis of their characteristics, they can be employed in different applications. For instance, Cao et al. reported the detection of small oligonucleotides (base pairs ranging from 2 to 10) using AeL without any additional modification of the generic experimental setup [43]. The ClyA nanopore, obtained from *Salmonella typhi*, has a larger pore size than other nanopores, therefore it found applications in large protein analysis and dsDNA translocation. Under ionic physiological conditions, DNA translocation cannot be performed due to the repulsion from native negative charges inside the pore. Further mutagenesis was employed to replace the negatively charged residues inside the pore lumen with positively charged residues, enabling DNA translocation to occur easily under physiological conditions [44,45]. Engineered MspA was studied for DNA sequencing and the detection of small molecules. Recently, phi29 DNA (DNAP) polymerase was employed to reduce DNA translocation speed, and clear current events were observed as DNAs passed through the engineered MspA nanopore, showing the possibility of an engineered MspA system for DNA sequencing [46,47]. Since many more potential applications of nanopores have been introduced to date, for interested readers, further detailed information regarding nanopore applications can be found in other excellent review papers [48,49,50].

Despite all these achievements, there still exist some hurdles before the commercial utilization of biological nanopores. The most important drawback of biological nanopores is the fragility of the housing material (lipid bilayer), which can be easily ruptured by high voltage, solution concentration, and mechanical perturbation. One alternative method is the use of solid-state nanopores. However, the uniformity of nanometer-sized pores is still limited and requires very expensive processes. The high noise-to-signal ratio due to the intrinsic properties of the materials is another problem to solve. Recent studies reported a novel approach to solve the problems by developing hybrid nanopores, which integrates a biological nanopore into a solid-state substrate. Hall et al. reported a hybrid nanopore that combines a genetically engineered α-HL nanopore with a silicon nitride (SiN) nanopore membrane [51]. Moreover, a DNA origami-graphene hybrid nanopore was also introduced for DNA sequencing [52]. These promising technologies open up an exciting possibility to advance an ultra-sensitive nanopore-based sequencing platform.

#### 2.1.2. Target-Specific Receptor-Mediated Biosensors

The biological olfactory system is known to have remarkable sensitivity and selectivity [58]. For example, honeybees show excellent discrimination ability that can distinguish a number of structurally similar molecules [59]. These properties are due to numerous olfactory receptors (ORs) in the olfactory sensory neurons (OSN). Entire ORs are G-protein coupled seven-transmembrane-helix proteins [60], in which the recognition of target molecules stimulates ion transfer through the TMP channel, resulting in depolarization of the OSN. Many researchers have proposed highly sensitive and selective biosensors by exploiting the mechanism of biological olfactory systems [60]. The OR-based sensing systems can be categorized into three kinds of platforms: whole cell, cell fragment, and artificial cell-based platforms.

**Figure 4:**
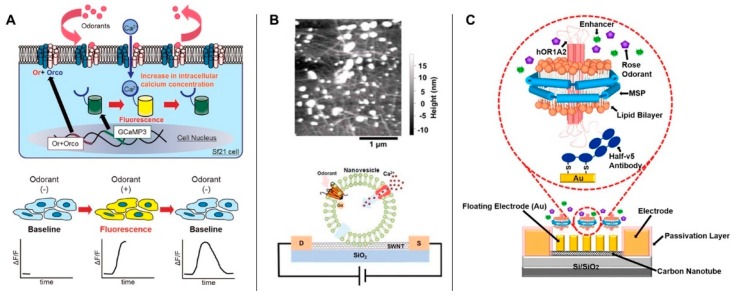
Olfactory receptor (OR)-based sensors. (**A**) Whole cell-based platform: odorant-stimulated ion transfer induces fluorescence illumination in the genetically modified cell [64]. (Reproduced with permission from Mitsuno et al. *Sci. Rep.,* 2015. Copyright© 2015 Elsevier) (**B**) Cell fragment-based platform: An olfactory system containing a cell fragment, an exosomenanovesicle, is immobilized onto the electric substrate [66]. Odorant-stimulated ion transfer induces impedance of the electric system. (Reproduced with permission from Ahn et al. *Sens. Actuator B-Chem.*, 2018. Copyright© 2018 Elsevier) (**C**) Artificial cell-based platform: An artificially constructed lipid membrane system, a nanodisc, is mounted with a purified olfactory system and immobilized onto an electric device [67]. The odorant-binding events of nanodiscs induce changes in the electric signal of the system. (Reproduced with permission from Lee et al. *Sci. Rep.*, 2018. Copyright© 2018 Nature Publishing Group).

In the whole cell-based platform, an engineered OR-encoded plasmid is transferred into living cells to synthesize and integrate desired ORs into the cell membranes. When target molecules specifically bind to the inserted ORs expressed in the cell, they induce ion transfer in the inner cellular space. There are a number of sensing systems that detect and quantify ion transport induced by the binding event of the ORs [61,62]. The Wang group exploited an electrical method to measure the binding events of the ORs [61]. They induced the synthesis and integration of ODR-10 (an OR from *C. elegans*) into the cell membrane. ODR-10 in living cells captured a specific organic compound, diacetyl, and induced ion transport, creating potential firing of the cell surface that resulted in transient potential changes. The potential firing was further recorded to quantify the diacetyl concentration. In addition to the electrical method, there are other assays to measure OR-based ion transfer through fluorescence [63,64] and surface plasmon resonance (SPR) [65]. For example, Mitsuno et al. established sf21 cell lines with insect ORs, Orco, and GCaMP3 as sensor elements [64]. When the odorant binds to Or and Ocro in the plasma membrane, they cause the influx of calcium ions that are bound with GCaMP3, showing fluorescence (Figure 4).

In a cell fragment-based platform, a necessary part of the cell, such as exosomes or nanovesicles, is utilized to build sensor systems. In general, fragments containing ORs are separated from whole cells and immobilized specifically onto a substrate. Then, as in the cell-based method, the binding events of the target molecules to the ORs are measured through various analytical methods, such as electrical methods, fluorescence methods, and SPR. 

In artificial cell-based platforms, ORs isolated from cells are inserted into artificially constructed lipid membranes such as liposomes and nanodiscs (Figure 4B,C). Briefly, Ahn et al. expressed olfactory receptors in a host cell and produced nanovesicles by simple agitation [66]. These nanovesicles were immobilized on a carbon nanotube and the binding of odorant was electrically detected. In the other study, Lee et al. used a nanodisc along with carbon nanotube-based field effect transistor system. Briefly, an hOR1A2-embedded nanodisc (hOR1A2NDs) was immobilized on the floating gold electrodes over a carbon nanotube-based transistor via thiol conjugation, which is called a protein-tethered bilayer lipid membrane (ptBLM) method. The binding events were electrically analyzed by underlying CNT based transistor [67]. 

Another artificial cell-based platform is a supported lipid bilayer (SLB), which is a self-assembled lipid membrane structure coated on a solid surface without chemical reaction. Hong et al. devised an SLB based odorant detection device [68]. In this device the OR integrated SLB is coated on an electric circuit consisting of CNT junctions and Au electrodes. When a target odorant binds to the specific OR, the conformational change of OR occurs, reducing the conductance of CNT junction. They reported that the device integrated with OR can measure the current change of electric circuit in real time and detect target odorant at femtomolar level. As such, the artificial cell-based platform often provides a highly controlled and sensitive system but lacks stability, while the whole cell-based platform provides a relatively stable system. 

Transmembrane protein based, or target specific receptor mediated, biosensors have received many attentions from various area such as food quality control [69], medical device [70], and environmental control [71]. To successfully apply highly sensitive TMP based biosensors to practical applications, understanding complex cellular function and increasing stability are the key to success. Many ongoing researches focus on developing more robust and reliable sensing systems.

#### 2.1.3. Acetylcholinesterase-Based Sensors

**Figure 5:**
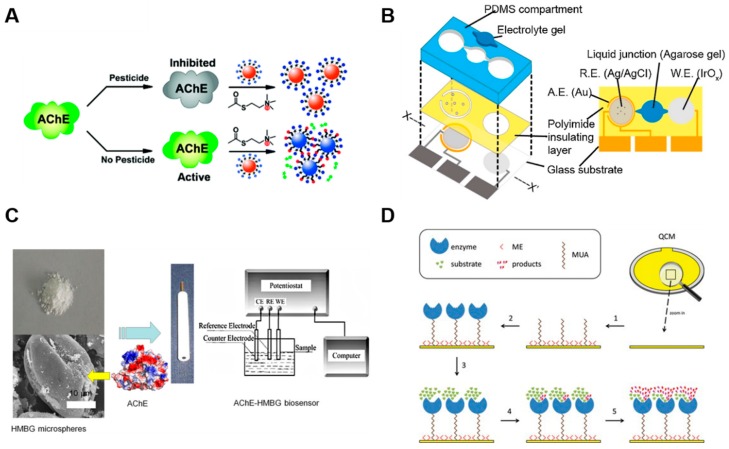
(**A**) A highly sensitive, rhodamine B-covered gold nanoparticle (RB-AuNP)-based assay with dual readouts (colorimetric and fluorometric) for detecting organophosphorus and carbamate pesticides in complex solutions [78]. (Reproduced with permission from Liu et al. *Anal. Chem.* 2012. Copyright© 2012 ACS publications) (**B**) Expanded view of the potentiometric biosensor device and a top view showing the mutual relationship between the structures of the layers [81]. (Reproduced with permission from Wang et al. *Sens. Actuator B-Chem.* 2015. Copyright© 2015 ACS Publications) (**C**) Schematic image of the acetylcholinesterase (AChE)-hierarchical mesoporous bioactive glass (HMBG) biosensor and an illustration of AChE adsorbed on the HMBG microsphere-coated electrode [82]. (Reproduced with permission from Lv et al. *Sens. J.Electron. Mater.*, 2017. Copyright© 2017 Springer nature) (**D**) Schematic image of the 11-mercaptoundecanoic acid (MUA), 2-mercaptoethanol (ME) and AChE enzyme coating process on a QCM-chip [86]. (Reproduced with permission from Bueno et al. *Anal. Let.*, 2013. Copyright© 2013 Taylor & Francis).

Acetylcholinesterase (AChE) is a part of the cholinergic nervous system that is found mainly at neuromuscular junctions and in chemical synapses of the cholinergic type. AChE is one of the most widely used TMPs for sensor systems. In particular, AChE can detect important neurotoxin compounds such as pesticides (carbofuran, malaoxon, malathion), Alzheimer’s disease drugs (donepezil, huperzine, galantamine, rivastigmine), chemical warfare agents (sarin, soman, tabun, VX), and natural toxins (aflatoxin, pyridostigmine) [72,73]. AChE-based sensors employ various types of transducers for their sensing systems, including optical, potentiometric, amperometric, and piezoelectric sensors. AChE can perform the indoxylacetate reaction, and the inhibition of AChE is therefore optically detected by measuring the production of indigo [74]. Chang et al. used maleimide functionalized tetraphenylethene and thiocholine reaction produced by AChE [75]. They made paper chip-type detectors and successfully detected pesticides, including diazinon, paraoxon, and malathion. The inhibition of AChE can also be detected by using a simple pH strip. AChE degrades acetylcholine into choline and acetic acid, which induces a color change in the acid-base indicator. Kostelnik et al. immobilized AChE onto commercially available pH strips with a gelatin membrane and evaluated the color change by using a smartphone [76]. Liu et al. and Sun et al. used gold nanoparticles to develop colorimetric sensors [77,78], while Hai et al. used quantum dots [79] (Figure 5A). A number of electrochemical biosensors have also been developed. Zhang et al. developed a potentiometric biosensor that measures the electrical potential of an electrode in a pH-sensitive membrane based on methylcellulose [80]. Similarly, Wang et al. detected organophosphate pesticides (OPs) based on sensing the pH change accompanying the enzymatic hydrolysis of OPs [81] (Figure 5B). Lv et al. synthesized three-dimensional hollow hierarchical mesoporous bioactive glass (HMBG) microspheres based on *Herba leonuri* pollen grains [82]. By establishing hierarchical porous structures, they successfully obtain a higher degree of adsorption of AChE. Through amperometric measurement functioning by the production of a current with an HMBG-coated electrode, malathion was detected (Figure 5C). Similarly, Ivanov et al. used carbon nanotube-coated electrodes to detect paraoxon and malaoxon, and Wang et al. used TiO_2_ nanoparticle-coated electrodes to detect carbaryl [83,84]. Piezoelectric biosensors measure affinity interactions between biodetectors and analytes. Quartz crystal microbalance (QCM) biosensors are the most widely used materials for piezoelectric biosensors due to their simple fabrication, good reproducibility, sensitivity, and low cost [85]. Bueno et al. used an AChE-embedded QCM sensor and detected the inhibition caused by physostigmine [86] (Figure 5D). Hossain et al. developed paraoxon and aflatoxin detection sensors by inkjet printing polyvinylamine and then overprinting AChE [87].

Butyrylcholinesterase (BChE), another cholinesterase, is expressed by the liver and detected predominantly in the blood plasma. It is also used in sensor systems to detect inhibitors such as pesticides. Arduini et al. developed an amperometric biosensor using BChE to detect paraoxon, a parasympathomimetic or pesticide [88]. They modified an electrode with carbon black nanoparticles (CBNPs) and immobilized BChE on it. Cho et al. a developed polyurethane-based ion-selective membrane and applied it in a BChE-based potentiometric sensor for paxagon detection [89]. White et al. detected tetraphenyl porphyrin by monitoring the change in the absorbance spectrum of AChE- or BChE-immobilized surfaces and compared their activity [74]. Similar to AChE, BChE can also be used in sensor systems as a bioreceptor. However, it has limited application compared to AChE due to its uncertain biological function [90]. Comparison between AChE-based sensors and BChE-based sensors are described in Table 2. AChE-based sensors provide real-time qualitative and quantitative information due to their fast response [91], while BChE-based sensors have low sensitivity. These problems have been being addressed by the development of nanomaterials, including CNTs, ZrO_2_ NPs, AuNPs, CdS NPs, and QCdS, and active research in related fields is ongoing [92].

### 2.2. Drug and Membrane/Membrane Protein Interaction Studies

#### 2.2.1. Small Molecule and Transmembrane Protein Interaction Studies

More than 50% of currently developed drugs, including Herceptin^®^ (trastuzumab) for breast cancer and Gleevec^®^ (Imatinib) for leukemia, target MPs [96,97], and almost 25% of the top 200 best-selling drugs target GPCRs [98]. Among the target MPs, GPCRs account for the highest percentage, up to 60%, followed by ion channels, receptors, enzymes, carriers, and transporters [99,100]. GPCRs are proteins that recognize a wide variety of ligands and transmit stimuli through a heterotrimeric GDP/GTP-binding protein into the cell. GPCRs account for over 25% of in vivo MPs, such as rhodopsin, epinephrine, and adrenaline. Since most of the important receptors in the body are GPCRs, they should be the main target of most drugs.

Currently, animal models are used for drug screening, which is not only time consuming, expensive, and unethical but also has variable results. Therefore, it is a very reasonable strategy to pursue alternative methods, such as an artificial membrane platform incorporating MPs, to analyze drug candidates. For this reason, preclinical testing using artificial membrane systems integrating TMPs has attracted increasing attention and is an actively developing field. The configurations of drug testing systems and sensor systems are very similar, as both are composed of a bioreceptor and a transducer, although they have different purposes [101]. A low noise amplifier for electrophysiological studies is a representative transducer of a TMP-based system. In vitro electrophysiological studies can be divided into whole cell measurements and artificial lipid membrane-based measurements. Whole cell electrophysiological measurements are performed by patch clamp tools, such as a Pasteur tip, or by placing cells on a micro/nano needle or a wide electrode after applying a drug to the target cell [102]. Whole cell electrophysiological measurements performed on the alpha subunit of a potassium ion channel (hERG) [97,102], neurotransmitter gamma-aminobutyric acid (GABA) [102], tryptophan synthase alpha chain (TRPA) [102], voltage-gated sodium channel (NaV) [103], or similar molecules [103]. Some TMPs can be obtained in sufficient quantities to be analyzed by artificial lipid membrane-based measurement, which has the advantage of enabling testing in a fully controlled environment with high-throughput screening. In this case, it is possible to obtain more precise information about the action mechanism of the drug because it can be measured at the single-molecule level. At present, electrophysiological screening has been performed on cystic fibrosis transmembrane conductance regulator (CFTR) [104], NaV [105], and hERG [106]. For example, Shaya et al. revealed that Mibefradil affects the pore domain by a protein dissection approach [105]. 

**Figure 6:**
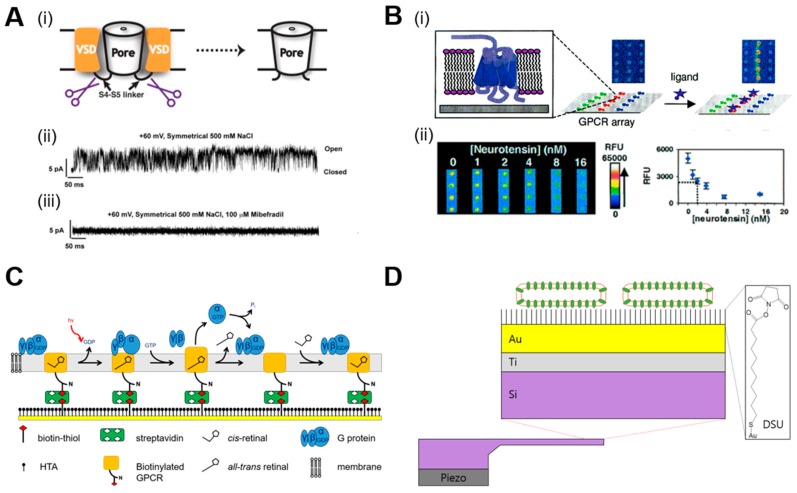
(**A**) (i) Cartoon depicting the strategy for creating a pore-only channel. Voltage-sensing domains (VSDs) are cut from pore domains. Bilayer recordings of the pore domain (Na_V_Sp1p) at +60 mV in symmetrical 500 mM NaCl before (ii) and after (iii) the addition of 100 μM mibefradil [105]. (Reproduced with permission from Shaya et al. *Proc. Natl. Acad. Sci.,* 2011. Copyright© 2011 NAS) (**B**) (i) Schematic of GPCR microarray. (ii) Fluorescence images and inhibition curve of arrays of the NTR1 receptor. The dotted line in the graph corresponds to the estimated IC50 value of ~2 nM [107]. (Reproduced with permission from Feng et al. *J. Am. Chem. Soc.*, 2002. Copyright© 2002 ACS Publications) (**C**) Carbohydrate-specific biotinylation of the latter results in uniform orientation of the bound receptor. The G protein binds to the supported lipid bilayer, which is formed after receptor immobilization [109]. (Reproduced with permission from Bieri et al. *Nat. Biotechnol.*, 1999. Copyright© 1999 Springer nature) (**D**) Schematic of the cantilever functionalization: the gold interface of the cantilever is prefunctionalized with a self-assembling DSU crosslinker, which binds to the gold via a thiol group and reacts by a succimidyl group with primary amines of FhuA–protein reconstituted in lipid vesicles [111]. (Reproduced with permission from Braun et al. *Nat. Nanotechnol.*, 2009. Copyright© 2009 Springer nature).

A, fluorescence molecule-based screening methods are also widely used to observe the mass transfer of TMPs via fluorophore quenching or luminescence, depending on the drug. Recently, Fang et al. developed a system for screening GPCR-incorporated model membranes on gamma-aminopropylsilane (GAPS)-coated surfaces [107] (Figure 6B). Dockendorff and Chris et al. produced K^+^ channel protein-embedded liposomes harboring fluorescent materials, and drug effects were compared by measuring the quenching rate of the fluorophore [108]. Similarly, fluorescence-based screening was performed to analyze Na^+^ channel proteins to determine the effects of anesthetic [104]. SPR is also widely used as a transducer. Bieri et al. coated gold with a biotin-thiol mixed self-assembled layer and bound the surface with a biotinylated receptor through a streptavidin-biotin reaction [109]. They measured the response of the GPCR depending on the change in the concentration of the agonist (Figure 6C). Patching SG is an example of an SPR-based TMP drug screening system and has been well described in another excellent review paper [110]. In addition, based on transducer phenomena, several other methodologies, such as cantilever [111] (Figure 6D) and thermal shift [112], have been used for drug screening and molecular interaction studies. Most MP-based screening platforms have limitations in incorporating the TMP into the analytical device with a complete 3D structure. Additionally, when the TMP contacts a solid substrate, it often loses its functionality or denatures. To overcome these limitations, various methodologies were employed, such as freestanding artificial membranes on small apertures [113], tethered lipid bilayer [114,115], or hydrogel cushioned lipid bilayer [116]; however, these techniques require long preparation time and high expertise in fabrication and handling due to lack of stability associated with a thin membrane [117,118]. Some researchers used polymer membranes instead of lipid membranes to increase the stability [119], but the stability of the polymer membranes were not greatly improved. The main drawback of a TMP-based system is that the membrane thickness of an amphiphilic region where TMPs are incorporated must be ~4–10 nm. These inherent limitations associated with the TMPs hinder developing membrane-based drug screening platforms.

#### 2.2.2. Small Molecule and Membrane/Membrane Protein Interaction Studies

**Figure 7:**
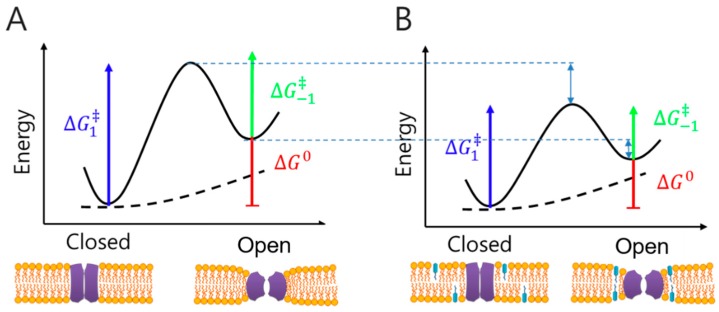
Free energy of membrane and membrane protein (**A**) without drug and (**B**) with drug. The drug alters the net free energy of the membrane by partitioning into the membrane.

Recent studies have shown that commercially available small molecules such as desipramine and mefloquine directly affect the membrane as well as MPs [120,121]. Accordingly, membrane and small molecule interactions can be quantitatively measured using a model MP as a molecular force probe [122]. TMPs, such as KcsA and mechano-sensitive MscS channels, cause structural changes and interact closely with the surrounding lipid bilayer [122]. The structural changes in TMPs perturb the lipid packing of the surrounding bilayer, and the deformation of the bilayer structure occurs with energy cost [99]. The difference in bilayer deformation energy associated with two protein conformations, “open” and “closed”, contributes to the total energy cost of the proteins’ conformational change. For this reason, when drug molecules are partitioned into the membrane, they greatly affect the operation of the MPs and the membrane stability due to the structural perturbation of the membrane. The magnitude of the drug effect varies depending on the degree of partitioning and the structural characteristics of the small molecules (Figure 7).

Gramicidin is the most widely used model MP for evaluating the effect of drugs or toxic molecules due to its simple structure and easy incorporation into the bilayer. When gramicidins from each leaflet of the bilayer are dimerized, an ion channel is formed, in which monovalent ions can pass through the channel, and deformation of the bilayer occurs in the surrounding lipid due to the thickness of gramicidin channels. The duration of the dimer is determined by the stability of the compressed bilayer. By measuring the duration of the dimer, the changes in the membrane properties induced by the partitioning drug can be measured. To quantify the effect of drugs or toxic molecules, ion current measurements and stopped flow-based fluorescence quenching methods were used for gramicidin-based membrane models. Previous researchers, including the Andersen group, analyzed interactions between membranes and diverse small molecules: detergents [123], alcohols [124], ionic liquids [125], phytochemicals [126], anesthetics [103,127], ions [128], and antimalarial compounds [129]. Because these membrane and small molecule studies give rapid and straightforward results and reveal molecular-level membrane property changes [122], this method can be used in early stage of drug development or discovery. A high-throughput artificial membrane platform will excel in the drug discovery process when available.

### 2.3. Energy Harvesting Using Membrane Proteins

Energy depletion and the development of alternative energy are persistent issues for humanity. Since the consumption of fossil fuel is steadily increasing, environmental problems, including air pollution, are emerging as more urgent issues than the depletion of fossil fuel [130,131]. Solar energy is always the most attractive source of clean energy, and researchers are always looking for more efficient methods to convert solar energy to heat or electricity. In particular, the energy conversion efficiency of an inorganic-based solar cell such as a conventional silicon material is reported to have a theoretical maximum of 33.7% [132]. However, biological systems have the most efficient components for the harvest of solar energy. MPs efficiently convert solar energy into different forms of energy to maintain cell homeostasis. Theoretically, these MPs have 100% energy conversion efficiency [133]. To mimic biological energy harvesting systems, researchers have exploited photoconverting proteins for in vitro energy conversion systems.

The energy conversion of photoconverter proteins is based on oriented electron or proton transfer among MPs [134]. Electrons are transferred through the electron donors and acceptors in the proteins (such as reaction centers), while protons are translocated by pigments, such as retinal pigments. Notably, the plants that utilize solar energy most efficiently contain chloroplasts that specialize in energy conversion in their leaves. The TMP complex within the plants is called a photosystem. Through electron transfer, the photosystem converts solar energy to another form of energy that can be used in vivo. Photosystems can be classified into photosystem I (PSI) and photosystem II (PSII) depending on the wavelength absorbed at the center where electron transfer starts, and there are certain differences between the position where the photosystems are mainly distributed and the detailed electron transfer cycle inside the chloroplasts.

PSI was first isolated in the 1960s, and its structure and function began to be investigated [135,136]. PSI is found not only in plants but also in algae and cyanobacteria, having the same function, but it is located in different structures depending on the host. A common plant PSI consists of a core complex and a light-harvesting complex (LHC). PSI contains 11–14 different polypeptides and several types of cofactors. The overall function of PSI involves harvesting photons and using their energy for electron transfer through a series of redox centers [137]. The electron transfer of PSI occurs as a cyclic reaction in which electrons originating from a representative central chlorophyll P700 eventually return to P700, during which NADPH is produced.

PSII is a complex mainly located in the thylakoid membranes [138]. PSII plays a crucial role in energy transfer by boosting an electron to a high energy level, resulting in water splitting that releases oxygen [139]. The overall structure is not significantly different from that of PSI, except for certain subunits, especially the chlorophyll at the center where electron transfer occurs, which reacts to a wavelength of 680 nm. In addition, PSII absorbs electrons from water molecules, and acyclic electron transfer is performed in which these electrons are finally transferred to PSI through PSII. This process forms a proton motive force (proton gradient) and produces ATP.

Among the MPs that can be used to produce energy by receiving solar energy, the rhodopsin family is found mainly in archaea [140]. Although there are rhodopsin families that occur in the retina of animals and participate in vision, this review focuses mainly on microbe rhodopsins that occur in the membranes of bacteria and use solar energy.

The most representative rhodopsin family involved in energy harvesting is bacteriorhodopsin (bR). bR is a proton pump found in the purple membrane of halobacterium [141] and generally occurs in the membrane as a trimer. When bR receives light, electron transfer occurs at the retinal chromophore of the bR center and transports protons across the membrane [142]. As a result, a proton gradient is formed at the boundary of the membrane, and this PMF is used in the production of ATP in vivo.

**Figure 8:**
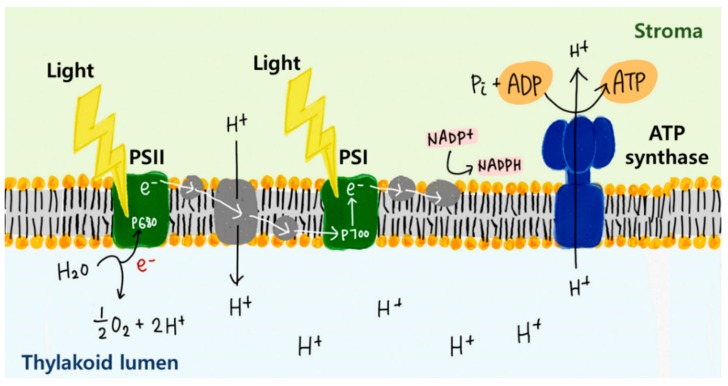
Schematic mechanism of photosynthesis in the thylakoid membrane (ATP production). When light reaches the reaction center of PSI and PSII, the excited electrons from the reaction center are transferred. At this time, a proton gradient is formed across the membrane due to the electron transfer and water splitting, and the proton concentration of the thylakoid lumen increases. Thus, the proton is transferred through ATP synthase from the thylakoid lumen to the stroma. As a result, ATP is synthesized at the stroma in the cell.

In general, ATP synthase is an enzyme that produces ATP, which is used as an energy source in vivo, and is a MP that exists in almost all organisms [143]. The most common ATP synthase consists of the F_0_ and F_1_ subunits, which are composed of a, b, c, and α, β, γ, δ, ε, respectively [144]. The F_0_ unit is responsible for fixing ATP synthase to the membrane, and the enzymatic site for actual ATP production/hydrolysis is the F_1_ unit [145]. When a proton gradient is established across the membrane where ATP synthase is present, the proton migrates through the ATP synthase [146,147]. The phosphorylation of ADP subsequently proceeds by proton transfer, producing ATP (Figure 8). The reversible hydrolysis of ATP also proceeds to maintain the proper ATP-ADP ratio in vivo. Each protein introduced in this section is summarized in Table 3.

**Figure 9:**
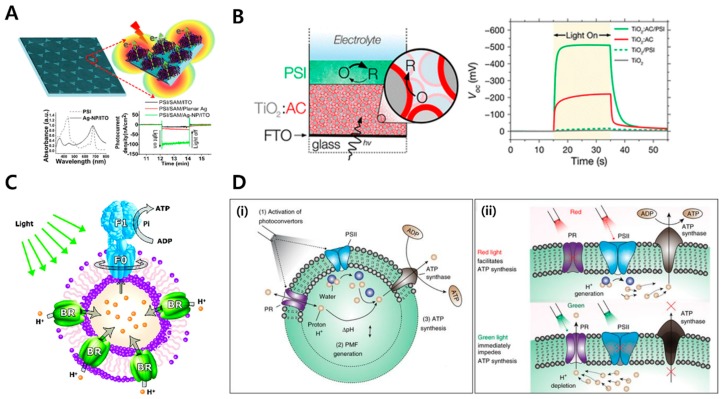
Energy harvesting application of transmembrane proteins. (**A**) Plasmon-enhanced photocurrent from photosystem I assembled on Ag nanopyramids [17]. With this system, the plasmon-enhanced photocurrents were enhanced up to ~6.5 and ~5.8 compared to PSI assembly on planar Ag substrates for wavelengths of 660 and 470 nm, respectively (Reproduced with permission from Pamu, R. et al. *J. Phys. Chem. Lett.*, 2018. Copyright 2018 American Chemical Society). (**B**) PSI multilayer film structure and increased photovoltage range [18]. A PSI multilayer film is assembled on a mesoporous film of TiO_2_ nanoparticles. The PSI multilayer promotes a more than 2-fold increase in photovoltage (Reproduced with permission from Robinson, M. T. et al. *ACS Appl. Energy Mater.*, 2018. Copyright 2018 American Chemical Society.). (**C**) Schematic representation of the proteopolymersome reconstituted with both bR and F_0_F_1_-ATP synthase [19]. When light reaches the bR, the proton transfers from outside to inside the proteopolymersome. Then, the generated proton gradient promotes ATP synthesis by ATP synthase. As a result, ATP is synthesized outside the proteopolymersome (Reproduced with permission from Choi and Montemagno, *Nano Lett.*, 2005. Copyright 2005 American Chemical Society). (**D**) Design and applications of the artificial photosynthetic organelle [20]. Using PR and PSII, ATP synthesis is controlled by different wavelengths of light. Red light facilitates ATP synthesis, while green light impedes ATP synthesis (Reproduced with permission from Lee, K. Y. et al. *Nat. Biotechnol.*, 2018. Copyright 2018 Springer Nature.).

As mentioned above, one can build a biomimetic photosynthesis system using TMPs to develop next-generation clean energy sources. An abiotic and biotic photovoltaic system combining inorganic and organic materials has been developed as a solution to overcome the theoretical limitations of photovoltaic systems using existing inorganic materials [161]. TMPs have been used to sensitize inorganic components (metals or semiconductors) [162,163]. In particular, PSI [148,149,150], PSII [151,152], and bR [153,154,155,156,157,158] have been combined with inorganic metal electrodes in a biohybrid photovoltaic system. Allam et al. demonstrated stable bR/TiO_2_ hybrid electrodes that can be used as photoanodes for photoelectrochemical water splitting. Under 100 mW/cm^2^ illumination, the bR/TiO_2_ hybrid electrodes achieved a photocurrent density of 0.65 mA/cm^2^. When they tested pure TiO_2_ nanotubes, the photocurrent density was 0.43 mA/cm^2^. Therefore, the photocurrent with TiO_2_ was increased by over ~50% [158]. LeBlanc et al. showed a highly successful biofunctionalization study with PSI-coated p-doped silicon electrodes [149]. A four-fold increased photocurrent density of 875 μA/cm^2^ was achieved compared to that of bare silicon. These studies showed the potential of the biohybrid photovoltaic system. More recently, studies with improved results have been reported [17,18,164,165,166]. Pamu et al. reported plasmon-induced photocurrent enhancements obtained from PSI immobilized on Fischer patterns of silver nanopyramids (Ag-NP) with enhancement factors of ~6 (Figure 9A). Robinson et al. demonstrated a new design for natural dye-sensitized solar cells containing PSI (Figure 9B). They stacked the photosynthesis assembly with a discrete PSI multilayer film atop a natural dye-sensitized photoanode. The stacked biohybrid photovoltaic system expanded the absorbance of the solar spectrum to facilitate more than a 2-fold increase in cell photovoltage compared to the unmodified equivalent.

In addition to applications to improve the efficiency of solar energy conversion, TMPs have been applied primarily to ATP production [167]. In vitro regeneration photosynthesis systems have been applied to artificial organelles or cells for artificial photosynthesis in the last two decades. Research on artificial cells has focused on ATP production by artificial photosynthetic systems, which may eventually provide the basic energy source of life, ATP, by themselves. Many in vivo mechanisms have not yet been investigated in vitro. Choi et al. demonstrated an artificial organelle in which ATP synthase produces ATP through a gradient of protons pumped by light by inserting a purified ATP synthase and bR from microorganisms into the triblock copolymersome (Figure 9C) [19]. The function of each protein was confirmed by the change in pH (bR) and the reaction of luciferase (ATP synthase). The proteopolymersome made of the two TMPs and ABA triblock copolymer showed a life span of 3–4 weeks. However, since the ATP produced in this system is outside the proteopolymersome, it has some limitations for use in other applications or more quantitative mechanistic studies, such as biochemical reactions within the proteoliposomes, as in real cells. Other studies have also attempted ATP production by combining PSII with ATP synthase in addition to bR [159]. Kuruma et al. demonstrated that the artificial system and the target proteins, including ATP synthase, can be directly produced through an in vitro system, such as advanced artificial cells [160]; however, the system showed that the ATP produced is still outside the liposomes, and the stability of the liposome was not studied. More recently, Lee et al. performed ATP synthesis using proteorhodopsin (PR) and ATP synthase and used the generated ATP for in vivo metabolism (Figure 9D) [20]. Giant unilamellar vesicles (GUVs) were used as the membrane platform, and purified target proteins from the recombinant host were encapsulated within the GUVs. While ATP was generated outside the liposome, as in previous studies, the liposome was once wrapped around the membrane to form multilayer vesicles, mimicking the plant cell structure, and ATP was harvested in the inner membranes to overcome the previous limitation. The ATP produced by this structure was used to carry out actin polymerization and carbon fixation continuously. Additionally, using proteorhodopsin (PR), which changes the direction of proton pumping according to pH, and PSII, dynamic reaction control was shown to accelerate or retard ATP synthesis depending on the wavelength of light and pH to be irradiated. Likewise, recent studies using MPs capable of energy harvesting have been expanding to research on artificial cells that can generate energy by themselves and consume energy directly.

For additional engineering applications, bR can be applied in a variety of electrical devices in industry, including batteries [168,169], memory [170], and biodefense devices [171,172,173]. In general, bR is difficult to isolate from the membrane and is therefore applied in the form of a purple membrane [174,175,176,177,178]. bR can be employed as the base material of microwave-absorbing paints for camouflage because bR exhibits strong microwave absorption (3–40 GHz). As a result, bR can be used for effective light diffraction and concealment for biodefense studies [171]. As such, TMPs capable of energy harvesting can have their own electrical properties and can be used as various biomaterials.

In the meantime, the maximum efficiency of the photosynthetic proteins has not yet been achieved, and the relatively short lifespan is the biggest problem in harvesting the amount of energy required by mankind. This short lifespan is due to the stability problem of the biological material itself. In the case of solar cells, the focus should be on techniques for more stable immobilization of TMPs on the electrode. In addition, photodamage directly to solar energy receivers may reduce the lifespan of the entire system. Several researches suggested that a self-assembly capable platform [179] can be adopted to reduce the photodamage and improve the efficiency of the photovoltaic system [180,181]. Ham et al. suggested a solar energy conversion system with high potential to overcome the photodamage using a reversible assembly process, showing high efficiency and long lifetime [181]. They focused on the only reaction center, not the whole protein. As a result, the photodamage was not extended to the whole protein. 

### 2.4. Water Purification with Membrane Proteins (Water Channels)

**Figure 10:**
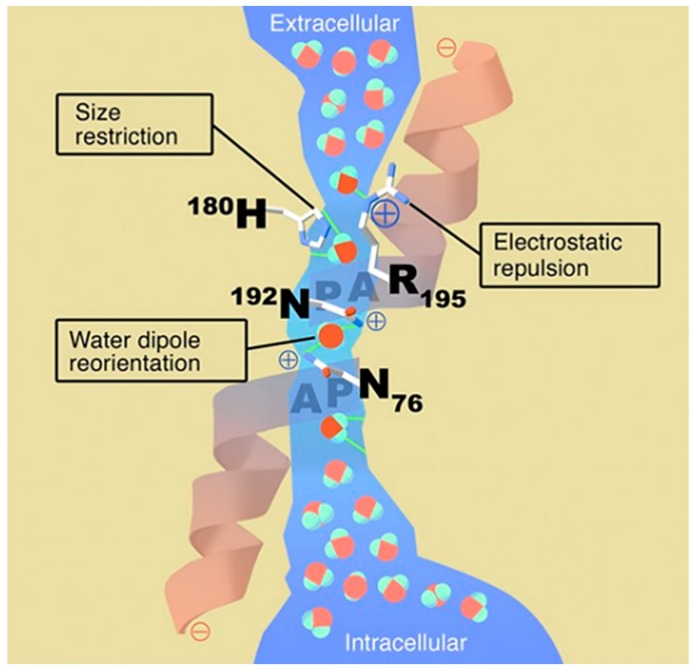
Aquaporin membrane structure with inter and extracellular vestibules containing bulk water and single file water molecule passage through a 20 Å span [184]. (Reproduced with the permission of Kozono et al. J. Clin. Invest. 2002, Copyright© 2002 American Society for Clinical Investigation).

In nature, water transport across cell membranes is facilitated by water channel proteins called aquaporins (AQPs); a major intrinsic protein (MIP) is present in all kinds of life. Aquaporin consists of two asparagine–proline–alanine (NPA) boxes that form intermolecular interactions with water molecules, creating water-selective pores. The narrowest diameter of the pore is 2.1–2.8 Å, which is approximately similar in size to a single water molecule. The electrostatic region in the channel prevents protons and other ions from passing through the channel and allows water molecules to pass in single file [182,183]. Figure 10 shows a schematic of water transport through the aquaporin protein [184]. Aquaporins are classified into three subfamilies depending upon their function and structure. Orthodox, or classical, aquaporin allows only water molecules to pass through [185], while aquaglyceroporin [186] also allows small molecules including glycerol, carbon dioxide, urea, and ammonia, as well as water molecules [187,188]. This family also contains the glycerol facilitator GlpF (glycerol permease facilitator), a glycerol-conducting channel. Both orthodox aquaporin and aquaglyceroporin have identical three-dimensional structures [189]. Finally, a recently discovered aquaporin family is known as “subcellular aquaporins” or “sip-like aquaporins” [190]. Currently, there are no clear functional studies reported except on water and glycerol permeation [191,192].

**Figure 11:**
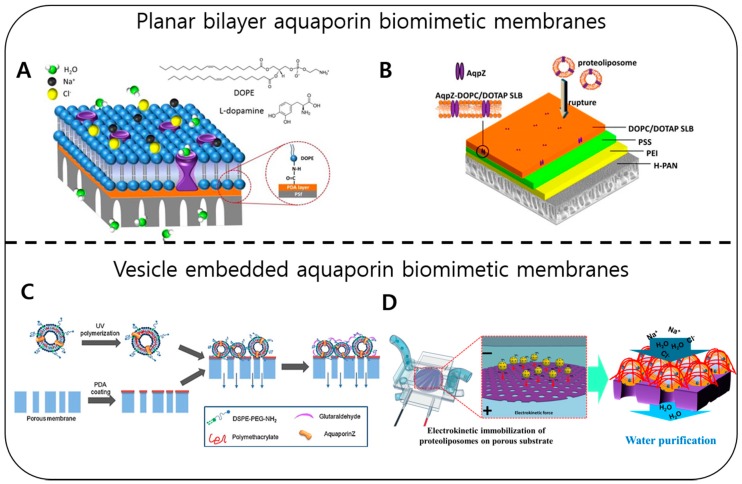
Shows the fabrication schemes of aquaporin biomimetic membranes. (**A**). Planar bilayer membrane fabrication using chemical conjugation. The bilayer attachment was performed by covalent bonding between the substrate and the lipid bilayer [195]. (Reproduced with the permission of Ding et al. *J. Mater. Chem. A,* 2015, Copyright© 2015 Royal Society of Chemistry) (**B**). Vesicle rupture on the functionalized substrate. The bilayer was formed by rupturing the vesicles on the polyelectrolyte functionalized substrate [196]. (Reproduced with the permission of Wang et al. *Environ. Sci. Technol*. 2015, Copyright© 2015 American Chemical Society) (**C**). Vesicle embedding using pressure. Vesicles were immobilized by applying negative pressure to the pores of membranes [197]. (Reproduced with permission from Sun et al. *Colloids Surf. B*, 2013, Copyrights© 2013 Elsevier) (**D**). Vesicle embedding using electrokinetic force. Under an applied electric current, the vesicles were immobilized on the voids of a solid substrate [16]. (Reproduced with permission from Fuwad et al. *Desalination*, 2019, Copyrights© 2019 Elsevier).

The in vitro incorporation of AQPs in a synthetic housing by Kumar et al. paved a path for biomimetic aquaporin membrane applications, showing the possibility of a water purification system. They calculated that the water permeability of aquaporin in the synthetic housing was 200-fold higher than that of commercial membranes [193]. Recently, a number of studies were reported to mimic the cellular water transport system for commercial water purification [194]. These aquaporin biomimetic membranes were fabricated in two different designs: (a) planar bilayer aquaporin biomimetic membranes and (b) vesicles containing aquaporin embedded in biomimetic membranes, as shown in Figure 11.

In both designs, the aquaporin proteins that provide a water-selective path were incorporated into an artificial housing, made of synthetic lipids or block copolymers. The aquaporin-containing vesicles were further stabilized on a porous solid substrate to enhance their mechanical stability for water purification applications. For planar aquaporin membrane fabrication, the aquaporin-containing vesicles were ruptured on porous solid substrates followed by a chemical or polymer coating [195,196] (Figure 11A,B). The ruptured vesicles consequently form a bilayer on the substrate covering all the vacant pores. However, this design shows incomplete coating of the substrate and membrane defects during water purification mainly because of the fragility of the bilayer.

The substrates used for immobilizing aquaporin-embedded vesicles provide additional stability and selectivity to the membranes. Because of the presence of selective layer, the aquaporin vesicles are protected from harsh external conditions, such as high hydraulic pressure; therefore, vesicle-embedded membrane substrate designs show better performance and enhanced stability than planar aquaporin membranes. Several methods have been developed to further enhance the stability and scalability of aquaporin membranes, including chemical adsorption [198], pressure-assisted vesicle adsorption [197] (Figure 11C), and chemical crosslinking [199], but defect-free aquaporin membrane fabrication without water leakage remains a challenge. Recently, Fuwad et al. presented a controllable methodology to uniformly coat aquaporin vesicles by electrokinetically driving aquaporin vesicles into the pores on the substrates, which gives better controllability during the coating process and enables high scalability without any harsh effects on the vesicles [16] (Figure 11D). Table 4 summarizes the aquaporin biomimetic membranes performance based on different designs.

Recent studies showed the promising future of aquaporin biomimetic membranes in water purification industry, as laboratory scale experiments demonstrated that aquaporin biomimetic membranes have high water purification performance [16], low fouling tendency [200], and high flux recovery [201] compared to conventional commercial membranes. Currently these aquaporin biomimetic membranes are being utilized and fabricated for pressure assisted water purification processes such as reverse osmosis (RO) and forward osmosis (FO). Continuous research and studies will reveal the other types of membranes which can be utilized for different application ranging from biomedical to personal healthcare like artificial kidney dialysis machine.

The successful implementation of commercial-grade biomimetic aquaporin membranes still faces several barriers and challenges, although the first aquaporin biomimetic membrane has been commercially launched by Aquaporin A/S [208]. Despite the superior performance of aquaporins [200,201,202], more study is required to understand the interaction of aquaporin protein with housing materials (lipids and polymers) to develop the best interfacing technology between abiotic and biotic components and to enable in vitro usability, which can be accomplished by overcoming the long-term stability problems and increasing the performance of membranes. The thermal stability and expensive MP purification (low yields) should also be addressed for the successful commercial realization of aquaporin membrane technology.

## 3. Conclusions and Future Perspectives

In this review, we have covered state-of-the-art biomimetic membrane applications with TMPs and their use in commercial-grade applications. MPs have unique functions in maintaining the homeostasis of living organisms; thus, MPs and their cellular functions are potentially the most attractive molecules as advanced substitutes for man-made systems. However, the representative applications of MPs discussed in this review utilize synthetic membranes as their housing material. The membrane not only protects the cellular entities as a barrier but also provides the unique environment TMPs require for their proper functions. Continuous advancements in protein engineering and purification technology are making it possible to obtain intact or functionalized TMPs for biohybrid applications, but the in vitro implementation of TMPs still faces serious challenges. Most importantly, TMPs are always embedded within housing materials (lipid/polymer membranes), which MPs require for proper folding and function. Therefore, the stability of housing materials is always the hurdle that must be overcome to take advantage of the unique functions of TMPs. Membrane engineering is critical for developing the MP applications mentioned in this review. Several substitutes are achieving success in replacing the gold standard lipidic housing with polymeric materials and nanolipoprotein particles (NLPs), which offers high throughput, stability, and increased control of the microenvironment during applications. In addition, the overexpression and yield of MPs also remains challenging, thereby hindering the tremendous potential of TMPS in practical applications. The conventional cell-based synthesis of MPs still involves hurdles in overexpression and consumes extensive time and effort, especially in the purification step. Cell-free protein synthesis (CFPS) methods can be an alternative solution to the problems associated with cell-based methods because CFPS can purify proteins in their original native environment without the use of detergent, which can affect protein folding and cause denaturation. Strong collaboration and collective research efforts from multidisciplinary communities, including membrane engineers, biologists, chemists, and nanotechnology specialists, are required to overcome the above problems and pave the path to ideal TMP biomimetic platforms with molecular-level responses and longevity. The continuous advancement in bionanotechnology may lead to a point where we can fabricate a complete biological cell through synthetic means, which will open a new world of TMP applications and revolutionize the biomedical industry by taking personal healthcare and disease diagnosis to the next level, enabling target cellular permeabilization, industrial/home water purification, efficient energy production systems, and DNA sequencing and detection.

## Figures and Tables

**Table 1:** Summary of the characteristics of biological nanopores including pore size, pore assembly, and their area of applications.

Nanopore	Pore Size (nm)	Channel Length	Applications	Pore Assembly	Reference
α-HL	1.4	Positive charge (pH 7.0)	RNA, dsDNA, ssDNA, proteins, small molecules	Heptameric	[26,53]
MspA	1.2	Negatively charged lumen	ssDNA, dsDNA	Octameric	[53,54]
ClyA	3.3	Negatively charged lumen	dsDNA, ClysA	Dodecameric	[45,55]
Ael	1.0	Positively charged lumen	ssDNA, proteins	Heptameric	[54]
Phi29	3.6	Positively charged lumen	ssDNA, dsDNA, large molecules and proteins	Dodecameric	[56,57]

**Table 2:** Summary of the various methods of detection.

Protein	Detection Method	Transducing Material	Analyte	Limit of Detection	Reference
AChE	Colorimetric	CS/DTNB	Methomyl, Profenofos	6.16 × 10^−4^ mM 0.27mM	[93]
AChE	Colorimetric	AuNP	Carbaryl Diazinon Malathion Phorate	0.1 μg/L 0.1 μg/L 0.3 μg/L 1 μg/L	[78]
AChE	Potentiometric	IrOx pH electrode	Potentiometric	1 μM	[81]
AChE	Potentiometric	CS/pH electrode	Malathion, Parathion-methyl, Methamidophos	0.6–600mM 0.1–100mM 0.1–100mM	[94]
AChE	Amperometric	AChE/CS@TiO2-CS/rGO/GC	Dichlorvos	0.036μM−22.6μM	[95]
AChE	Amperometric	Glass microsphere	Malathion	0.0135 ppb	[82]
AChE	Piezoelectric	ME/MUA/QCM	Physostigmine	1 mg/mL	[86]
BChE	Amperometric	CBNPs	Paraoxon	5 μg/L	[88]
BChE	Potentiometric	PU/HPU	paraoxon	10 nM	[89]

**Table 3:** Summary of the characteristics of energy harvesting proteins including their function, application, and the novelty and limitation of application.

Protein	Function	Application	Novelty of Application	Limitation of Application		Reference
				In Common	Each	
PSI	Light absorption (700 nm) and Electron transfer	Solar energy harvesting (e.g., Biohybrid photovoltaic system)	Clean energy source, the highest theoretical efficiency	Short lifespan, difficulty of achieve maximum theoretical efficiency	Hard to recover when photodamage occurs (almost degraded)	[17,18,148,149,150]
PSII	Light absorption (680 nm) and Electron transfer				Photoinhibition of PSII under strong light reduces the activity.	[151,152]
bR	Proton pumping				Difficulty in overexpression	[153,154,155,156,157,158]
ATP synthase	ATP synthesis	ATP production	Artificial cell research		Low activity at low temperature	[19,20,159,160]

**Table 4:** Summarizes the performance of aquaporin biomimetic membranes based on their two designs.

Membrane Design	Protein Housing	Fabrication Techniques	Membrane Performance	Limitations	References
Vesicles embedding	Synthetic lipids	Electrokinetic force, pressure force, magnetic adsorption	3.6–55.2 LMH * 20%–97.8 ± 0.7% salt rejection	Small membrane size, harsh coating conditions, defects in large area membrane	[16,199,202,203]
	Synthetic polymers	Adsorption chemical bonding, pressure assisted coating	16.4–22.6 LMH 61%–90% salt rejection	No uniform surface coating, low water desalination property shows high defects in coating	[204,205,206]
Planar bilayer	Synthetic lipids	Chemical bonding, vesicles fusion via electrostatic interactions	5.5–6.31 LMH 61%–90% salt rejection	Incomplete surface coating, unstable under high pressure, low performance	[195,196]
	Synthetic polymers	Vesicles rupturing with disulfide-gold conjugation	8.2 LMH/bar 45.1% salt rejection	Low water purification, incomplete surface coating	[207]

* It is water permeability (LMH= Liter per meter square per hour).
